# Rapid preparation of polydopamine coating as a multifunctional hair dye†

**DOI:** 10.1039/c9ra03177d

**Published:** 2019-07-02

**Authors:** Zhong Feng Gao, Xin Yu Wang, Jian Bang Gao, Fan Xia

**Affiliations:** Collaborative Innovation Center of Tumor Marker Detection Technology, Equipment and Diagnosis-Therapy Integration in Universities of Shandong, Shandong Province Key Laboratory of Detection Technology for Tumor Markers, School of Chemistry and Chemical Engineering, Linyi University Linyi 276005 China gaozhongfeng@lyu.edu.cn; Department of Natural Science, Linyi University Feixian Campus Linyi 273400 China; Engineering Research Center of Nano-Geomaterials of Ministry of Education, Faculty of Material Science and Chemistry, China University of Geosciences Wuhan 430074 China xiafan@cug.edu.cn; Hubei Key Laboratory of Bioinorganic Chemistry & Materia Medica, School of Chemistry and Chemical Engineering, Huazhong University of Science and Technology Wuhan 430074 China

## Abstract

Dyeing of hair is an interesting research field within the cosmetics industry due to the increasingly aging population worldwide. In order to reduce the toxicity of hair dye materials and improve the speed of hair dyeing, we developed an *in situ* polymerization of dopamine catalyzed by copper sulfate and hydrogen peroxide on the hair surface to form a polydopamine (PDA) coating for hair dyeing. The morphology and elements of polydopamine on hair were characterized. The durability, thermal insulation, and bacteriostasis performance of PDA hair dye were discussed. The results showed that human hair can be dyed by PDA in as little as 5 min with comparable dyeing results to those of commercial products. PDA-based hair dye displayed significant durability, and barely faded after continuous washing with shampoo (30 times). After PDA dyeing, the thermal insulation performance was enhanced, which could prevent external heat invasion in summer and local heat dissipation in winter, increasing the level of comfort. In addition, remarkable antibacterial properties were demonstrated, which could effectively prevent the occurrence of bacterial inflammation on the scalp. These results might push forward the evolution of nanomaterial-based hair dyes with promising green, healthy, and user-friendly advantages.

## Abstract 2

A rapid and effective polydopamine-based method for dyeing human hair was demonstrated, which achieved a significant black color, remarkable durability, enhanced thermal insulation performance, and anti-bacterial property.

## Graphical abstract

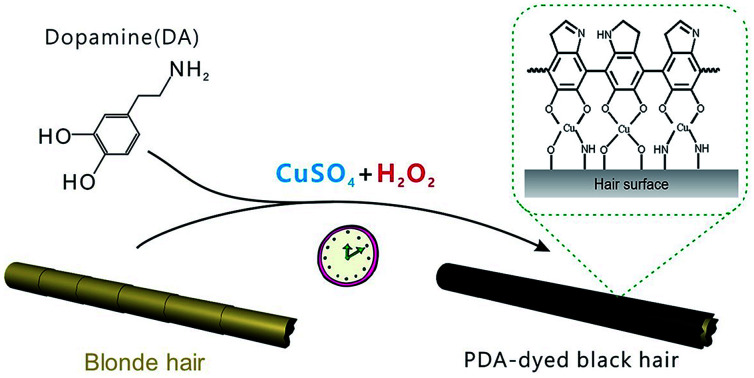

Hair dyes are widely used for enhancing the appearance of hair or as a fashion statement. Natural hair color is determined by the presence of the pigment melanin in the cortex and medulla, and the breakdown of melanin leads to gray or white hair.^1^ Since dark to black-colored hair is the most common color worldwide, there is great demand for similarly colored hair dyes. Modern commercial hair dyes permanently color the hair by a series of oxidative, dye-forming reactions with an alkali, usually ammonia.^1,2^ Small diamine-based organic species, including *para*-phenylenediamine (PPD) and its derivatives, have been widely used as permanent hair dyes.^3^ Oxidants, almost exclusively hydrogen peroxide, allow diamine compounds to copolymerize with couplers (*e.g.*, *m*-phenylenediamines, resorcinol, naphthols, and their derivatives), producing various colors.^4^ However, organic compounds with molecular masses of less than 500 Da, such as PPD, have been shown to easily cross the skin barrier and are regarded as human allergens and carcinogens.^5^

To address this issue, some nontoxic nanomaterials, which are versatile in biomedical,^6–8^ nano-engineering,^9,10^ and clean energy^11,12^ applications, have also been developed as safe dyeing agents. For example, Huang and coworkers found that graphene-based hair dyes could greatly shorten the dyeing time and improve antistatic performance.^2^ Functionalized carbon nanotubes and carbon particles have been used to dye hair black by forming a uniform and thin surface coating.^13,14^ Using melanin extracted from biological sources as a hair dye is another option because melanin is an *in vivo* hair color agent in humans.^15,16^ Polydopamine (PDA), a black pigment naturally occurring in melanin, has been demonstrated to deposit on virtually all types of materials, including inorganic and organic surfaces, with high adhesiveness and a controllable thickness.^17–19^ PDA also exhibits many remarkable features in electrical, optical, and heat transfer applications as well as striking biocompatibility.^20–23^ However, few reports have focused on the black color of PDA, which is an attractive property for applications in cosmetics.

Here, we report a PDA-based hair dye that achieved PDA deposition on hair surfaces using CuSO_4_/H_2_O_2_ as a trigger. This method afforded comparable results and faster reaction times (∼5 min) than those of commercial permanent hair dyes. Due to the strong adhesion of PDA, the color of PDA-dyed hair faded only slightly after 30 washes, indicating significant stability. The deposition of PDA on the hair surface enhanced the warmth retention of the hair. In addition, the deposited layer exhibited excellent antibacterial properties due to the involvement of copper ions. This report provides a rapid, multifunctional PDA-based hair dye, which is critical for developing hair dyes based on novel nanomaterials and has high practicality and potential marketability.

The typical dye solutions contained DA as the monomer and CuSO_4_/H_2_O_2_ as the trigger. CuSO_4_ and H_2_O_2_ could rapidly induce the deposition of the PDA coating onto the hair surface (Fig. 1). In this reaction, reactive oxygen species (ROS), such as HO_2_˙, O_2_˙^−^, and OH˙, are produced by mixing Cu^2+^ and H_2_O_2_ in an alkaline medium.^23^ The resulting radicals greatly facilitate the polymerization of DA and increase the deposition rate of the PDA coating.^24^ The main component of hair is protein, allowing extensive carboxyl, amine, and hydroxyl groups on the hair surface.^2,25^ Previous literatures demonstrated these functional groups displayed high binding affinity for copper ions.^26–28^ Thus, PDA could be initially generated on the hair surface to form a thin layer. The PDA products chelated to each other by copper ions *via* the layer-by-layer deposition, producing the final PDA coatings. The gel permeation chromatography (GPC) was employed to measure the molecular weights (*M*_w_). As shown in Fig. S1 (ESI†), there are two peaks that locate around 550 Da and 1690 Da in the GPC curve, which might be derived from different polymerization mechanisms, including self-polymerization and Cu^2+^-coordinated polymerization.^29,30^Fig. 2a plays the color change and UV/Vis absorbance for various DA solutions. The DA solution with CuSO_4_ and H_2_O_2_ changed significantly to a black color. When CuSO_4_ or H_2_O_2_ were present independently, the color change was quite small. A characteristic peak at 465 nm was present in the UV/Vis spectra (Fig. 2b), and this peak was attributed to the product of PDA.^23^ The reaction kinetics were investigated by recording the UV/Vis absorbance at 465 nm. As shown in Fig. S2 (ESI†), the absorbance reached a plateau within 5 min, indicating a rapid reaction process.

**Fig. 1:**
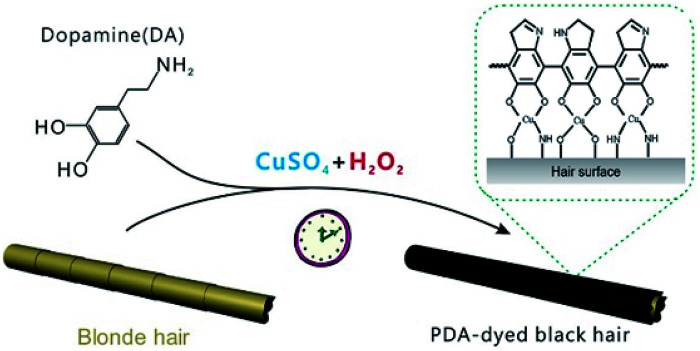
Schematic of the blonde hair dyed with a PDA coating.

**Fig. 2:**
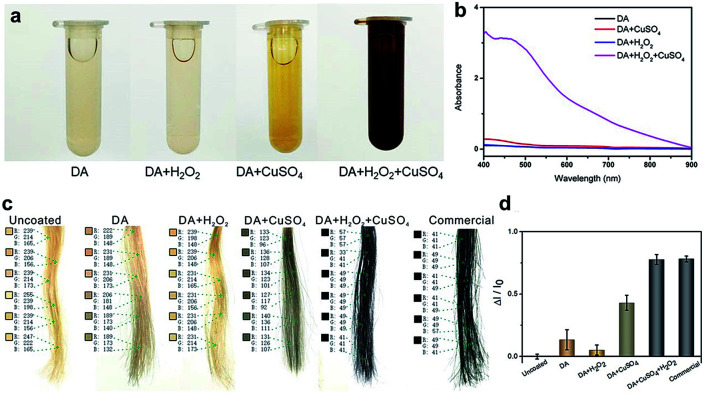
(a) The color change and (b) UV/Vis absorbance spectra of various DA solutions. (c) The digital picture and (d) variations in the RGB intensity of uncoated hair, DA dyed, DA + H_2_O_2_ dyed, DA + CuSO_4_ dyed, DA + H_2_O_2_ + CuSO_4_ dyed, and commercial product-dyed hair.

The beneficial functional groups, such as catechol, amine, and imine groups, in PDA can act as both anchors for the loading of transition metal ions and starting points for covalent modification with desired fragments.^31,32^ With these advantages, PDA is, unsurprisingly, capable of binding to hair surfaces with the assistance of metal ions. To test the performance of PDA hair dye, blonde hair samples were used. As shown in Fig. 2c, a black color can be generated by adding CuSO_4_ and H_2_O_2_. For quantitative analysis, the color intensity was first determined using Adobe Photoshop software (version CS 6). We randomly selected six points in the digital image and computed the RGB average value (*I* = *I*_R_ + *I*_G_ + *I*_B_). Blonde hair served as the background of the control experiment (*I*_0_). The adjusted intensity (Δ*I*) was calculated as Δ*I* = *I* − *I*_0_. Thus, the variation rate of the intensity was defined as Δ*I*/*I*_0_. As shown in Fig. 2d, the intensity of DA with CuSO_4_/H_2_O_2_ changed significantly (77.6%), and this variability is comparable to that of commercial products (78.1%). In addition, it displayed a much higher rate of change than solutions of dopamine alone (13.5%), dopamine with H_2_O_2_ (5.2%) and dopamine with CuSO4 (43%).

As shown in Fig. S3a (ESI†), the morphology of the hair surface was characterized by SEM. The hair surface was observed to be uniformly coated with a layer of PDA and no obvious aggregation or scaling was present. The SEM-EDS mapping images confirmed that PDA-chelated Cu^2+^ was well-distributed and anchored on the hair surface (Fig. S3b, ESI†). Inductively coupled plasma mass spectrometry (ICP-MS) was used to quantify the concentration of copper ions. We found that the concentration of Cu^2+^ increased from 12.7 mg kg^−1^ to 5306.6 mg kg^−1^ after the dyeing procedure completed.

To achieve the best performance, the experimental conditions, such as the concentrations of DA, CuSO_4_, and H_2_O_2_ and the reaction time, were optimized.

The absorbance of PDA was investigated based on the UV/Vis spectra with various DA concentrations (Fig. S4, ESI†). The absorbance intensity increased with increasing concentrations of DA from 0.1 mg mL^−1^ to 1 mg mL^−1^. When the DA concentration was further increased, the absorbance changed only slightly, indicating the saturation point had been reached. Thus, 1 mg mL^−1^ DA was the minimum concentration for conducting the reaction.

The concentrations of CuSO_4_ and H_2_O_2_ were optimized, and the results are displayed in Fig. S5 and S6 (ESI†), respectively. Slight changes of the absorbance were observed below 10 mM CuSO_4_ and 15 mM H_2_O_2_. When we further increased these concentrations, the absorbance changed only slightly. The obtained data demonstrated that at least 10 mM CuSO_4_ and 15 mM H_2_O_2_ should be used.

The dyeing time after the addition of all reagents was investigated. We recorded the change in the color (Δ*I*/*I*_0_) at room temperature over 15 min (Fig. 3). The calculated relationship of Δ*I*/*I*_0_*vs.* time is shown in Fig. S7 (ESI†). We observed that Δ*I*/*I*_0_ rapidly increased in approximately 5 min and then remained stable, indicating that the proposed method has remarkable simplicity and rapidity.

**Fig. 3:**
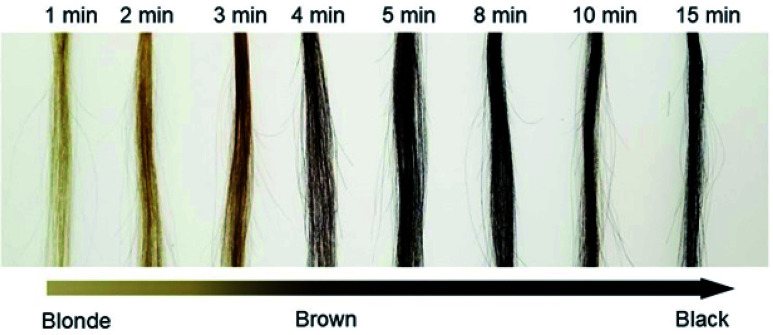
The digital images show the result of PDA hair dye with different dyeing times.

To test the durability of the PDA coating, elaborate washing tests on hair treated with PDA were conducted using shampoo. As shown in Fig. 4, the PDA coating strongly adheres to hair and cannot be easily washed off by washing 30 times with shampoo, and this result was quantitatively confirmed by chromaticity analysis. This durability can be attributed to the following deposition mechanisms. Keratin, the key protein at the surface of hair, contains carboxyl, amino and hydroxyl groups, which can strongly bind with DA under alkaline condition.^2,25^ The copper-assisted cross-linking could trigger the polymerization of dopamine with keratin, generating a thin layer of PDA on the hair surface. Then, deposition continued in a layer-by-layer manner with the copper ions chelating the PDA products to each other. In our reaction conditions, the inherent adhesiveness of the PDA independent of copper ion assistance was demonstrated to contribute to the coloring of the hair. Notably, the hair surface should be clean enough before treatment. Because the grease and other contaminants may weaken the strength between PDA with substrate.^33^

**Fig. 4:**
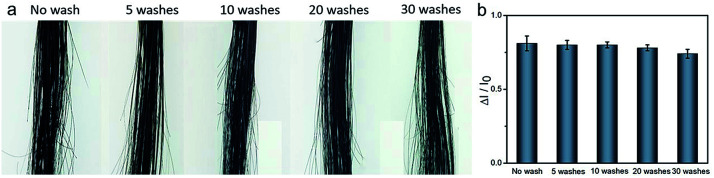
(a) The digital images show the PDA-coated hair before and after repeated washings with shampoo. (b) The corresponding RGB color variations.

Hair plays a critical role in regulating the body temperature of intricate biological systems. Improving the thermal insulation performance of hair is a means of promoting its thermoregulation performance. For example, lower heat dissipation from the head could increase comfort in winter. To test its thermal insulation performance, the heating and cooling rates of PDA-coated hair were compared with those of uncoated hair and hair treated with a commercial dye. All samples were first kept at room temperature (25 °C) and then brought into contact with a heating plate set at 40 °C. An infrared (IR) camera was used to take digital images during heating. As shown in Fig. 5, a lower overall temperature increase was observed for PDA-coated hair, which was ∼10 °C cooler than the other two samples within the first 4 s of heating. To test the cooling rates of PDA-coated hair, all hair samples were preheated to 40 °C and then removed from the heating plate. Images were acquired with an IR camera, and the results revealed that the PDA-coated hair samples dissipated heat at a lower rate. The overall temperature of the PDA-coated hair was already more than 5 °C warmer than both the hair treated with commercial dye and the uncoated hair within 4 s of cooling, and it reached room temperature more slowly. The difference in temperature is sufficiently large such that it can be felt by human skin, preventing external heat invasion in summer and local heat dissipation in winter, increasing the level of comfort.

**Fig. 5:**
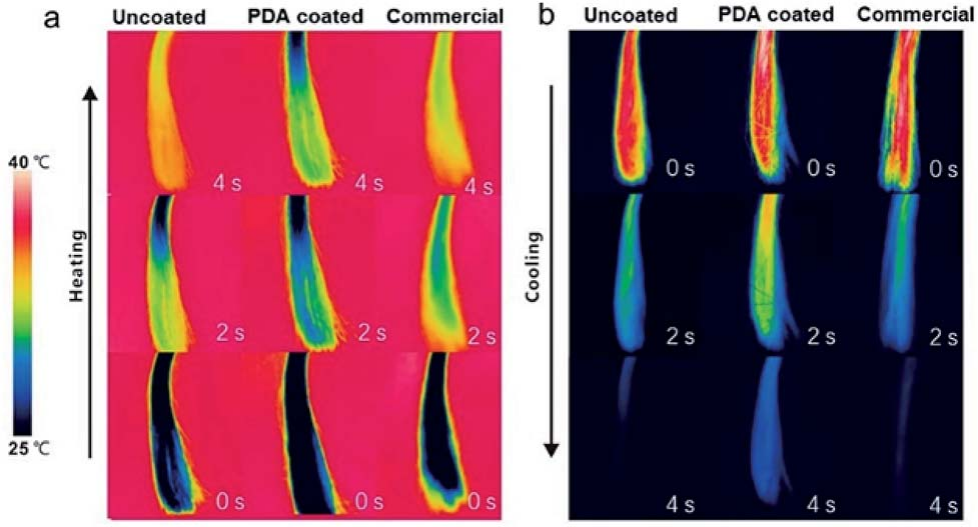
Infrared thermal images show the thermal profiles of uncoated hair, PDA-coated hair, and commercial product-dyed hair during (a) heating and (b) cooling.

To demonstrate its biocompatibility, the tetrazolium (MTT) based assays were conducted to evaluate the cytotoxicity of the PDA to human skin fibroblasts (HSF). The experiment was conducted following 0.5 h and 24 h incubation time (Fig. 6a). Significant cell viability reduction was observed after 24 h incubation time. Toxicity results of PDA in HSF cells indicated a IC_50_ value at 45.7 μg mL^−1^. However, the cell viability was estimated to be greater than 99% with 1–200 μg mL^−1^ of PDA for 0.5 h. As the retention time of PDA does not exceed 0.5 h on scalp, the proposed material shows significant biocompatibility. Next, rabbit skin irritation test was performed to evaluate the safety of PDA material on skin. As shown in Fig. 6b–e, there was no edema or erythema in the ear and back skin of rabbit, respectively. Taken together, our proposed material is biocompatible, healthy, and promising for practical application.

**Fig. 6:**
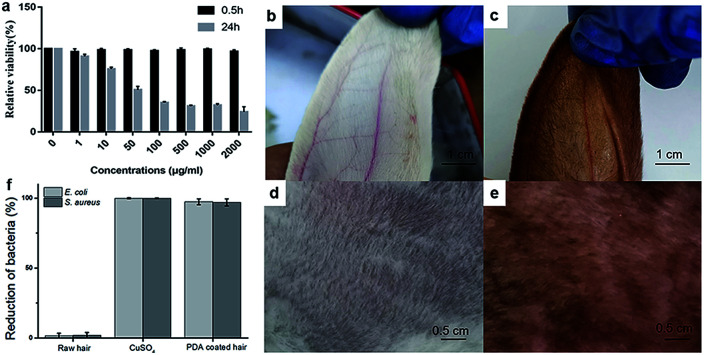
(a) Cellular toxicity of PDA to HSF cells. The rabbit skin irritation test on ear (b, before dyeing; c, after dyeing) and back (d, before dyeing; e, after dyeing). (f) Antibacterial performances of raw hair, CuSO_4_, and PDA coated hair, respectively.

Similar to Ag, copper ions also exhibit significant antibacterial activities toward various bacterial strains, such as *E. coli* and *S. aureus* (Fig. 6f). As mentioned above, copper ions can chelate PDA to facilitate polymerization, providing a PDA coating with excellent antibacterial performance toward *E. coli* and *S. aureus*. As shown in Fig. 6f, ∼97.4% of *E. coli* growth and 99.9% of *S. aureus* growth were inhibited during the antibacterial assay, while the uncoated raw hair continued to show bacterial growth. The antibacterial performance of PDA allows it to prevent bacterial dermatitis on scalp and enhance the quality of daily life.

## Conclusions

In conclusion, a rapid and effective PDA-based method for dyeing human hair was developed. PDA coatings produced by copper ions and hydrogen peroxide achieved a significant black color on human hair, similar to what is achieved with commercial hair dyes. Moreover, this treatment could be accomplished in ∼5 min, which is faster than what is possible with commercial products. Owing to the inherent adhesive nature of PDA, we demonstrated that this PDA-based hair dye shows strong adhesion, as it fades only slightly in 30 washes with shampoo. The PDA coating on the hair surface enhanced its thermal insulation performance, which might increase the level of comfort. In addition, the PDA-based hair dye exhibited remarkable antibacterial performance, which might prevent bacterial dermatitis on scalp and enhance the quality of daily life. Taken together, these results demonstrate that the PDA-based hair dyes are comparable to commercial hair dyes and exhibits some superior characteristics, such as enhanced thermal insulation and antibacterial performances. We expect this PDA-based hair dying method to be extended to other practical applications, including energy storage materials or sensing devices.

## Conflicts of interest

There are no conflicts to declare.

## Supplementary Material

RA-009-C9RA03177D-s001
