# Supplementary material for: Rapid preparation of polydopamine coating as a multifunctional hair dye
Source: RSC Adv. 2019 Jul 2;9(35):20492–6. doi: 10.1039/c9ra03177d (PMC9065481; doi:10.1039/c9ra03177d)
Supplement: RA-009-C9RA03177D-s001 [file RA-009-C9RA03177D-s001.pdf]

Electronic Supplementary Material (ESI) for RSC Advances.
This journal is © The Royal Society of Chemistry 2019

Electronic supplementary information

Rapid preparation of polydopamine coating as a multifunctional hair dye

Zhong Feng Gao[*a], *Xin Yu Wang*[a], *Jian Bang Gao*[b], *and Fan Xia*[*cd]

[a] Collaborative Innovation Center of Tumor Marker Detection Technology, Equipment and Diagnosis-Therapy Integration in Universities of Shandong, Shandong Province Key Laboratory of Detection Technology for Tumor Makers, School of Chemistry and Chemical Engineering, Linyi University, Linyi 276005, China.

[b] Department of Natural Science, Linyi University, Feixian Campus, Linyi 273400, China.

[c] Engineering Research Center of Nano-Geomaterials of Ministry of Education, Faculty of Material Science and Chemistry, China University of Geosciences, Wuhan 430074, China.

[d] Hubei Key Laboratory of Bioinorganic Chemistry & Materia Medica, School of Chemistry and Chemical Engineering, Huazhong University of Science and Technology, Wuhan 430074, China

Correspondence: Z. F. Gao (E-mail: gaozhongfeng@lyu.edu.cn) and F. Xia (E-mail: xiafan@cug.edu.cn)

Experimental Section

Materials: Dopamine was purchased from Aladdin Chemical Co. Ltd, China. E. coli and S. aureus were purchased from Guangdong Microbial Culture Collection Center. Phosphate buffer solution (PBS) was purchased from Sangon Biotechnology Co. Ltd, China. Blonde hair was purchased from Amazon (Emosa, #60 platinum blonde). Commercial hair dye from Joybuy (Swarovski, 2.0 Pearl black). Shampoo from Joybuy (Qingyang, multi-effect water moisturizing maintenance). Other reagents, such as Hydrogen peroxide (30%, H_2O_2), copper sulfate pentahydrate ($CuSO_4 . 5H_2O$), were purchased from Sinopharm Chemical Reagent Co., Ltd and used without further purification. Water used in all experiments was deionized and ultrafiltrated to 18.2 MΩ.

Hair dyeing with polydopamine: The hair sample were first washed with water thoroughly to eliminate impurities on the surface. Then, add the fresh prepared mixture contained with dopamine (5 mg/mL), Cu^{2+} (10 mM), and H_2O_2 (15 mM). The entire dyeing process can be completed about 5 min. Then, the dyed hair was washed and dried in air.

Durability test of PDA hair dye: The hair dyed with PDA was immersed in 5 vol% of shampoo in 40 mL of water in a 50 mL centrifugation tube. Then, the tube was vigorously shaken for 5 min on a vortex mixer. After washing, the hair was cleaned with water and dried in air. The PDA coating can endure over 30 washes without obvious decoloration.

Antibacterial test of PDA hair dye: First, the crude or PDA-dyed hair was placed into a 24-well culture plate. A 200 μL of E. coli or S. aureus suspension in PBS (1×10^7 cfu/mL) was used to cover the substrate. Add 1800 μL of PBS to dilute the bacterial solution after 2.5 h at 37 ℃ for agar plate incubation. Then, the bacterial suspension and each sample were transferred to a new tube for 5 min ultrasonic treatment to detach the adhered bacteria. Finally, the bacterial solution was diluted to 1000 times with PBS solution and 100 μL of the bacterial solution was taken to measure the viability of bacterial by using agar plates.

Characterization: Morphology of the samples was observed by scanning electron microscope (SEM, QUANTA250, USA). X-ray photoelectron spectra were collected by a spectrometer (XPS, Perkin Elmer, USA) with Al Kα excitation radiation (1486.6 eV). UV-vis absorption was tested with an ultraviolet spectro-photometer (UV 2450, Shimadzu, Japan). MTT assays were performed on a BioTek Epoch2 microplate reader. Inductively coupled plasma mass spectroscopy (ICP-MS) were conducted on an ICPOES730 instrument (Agilent). Gel permeation chromatography (GPC) was carried out with a Shimadzu LC-10AD (column, TSKgel GMPWXL; solvent, water; flow rate 0.6 mL/min; 35 ℃). The digital images were taken on a shadow-less plate by a camera (5D Mark II, Canon) in a professional studio.

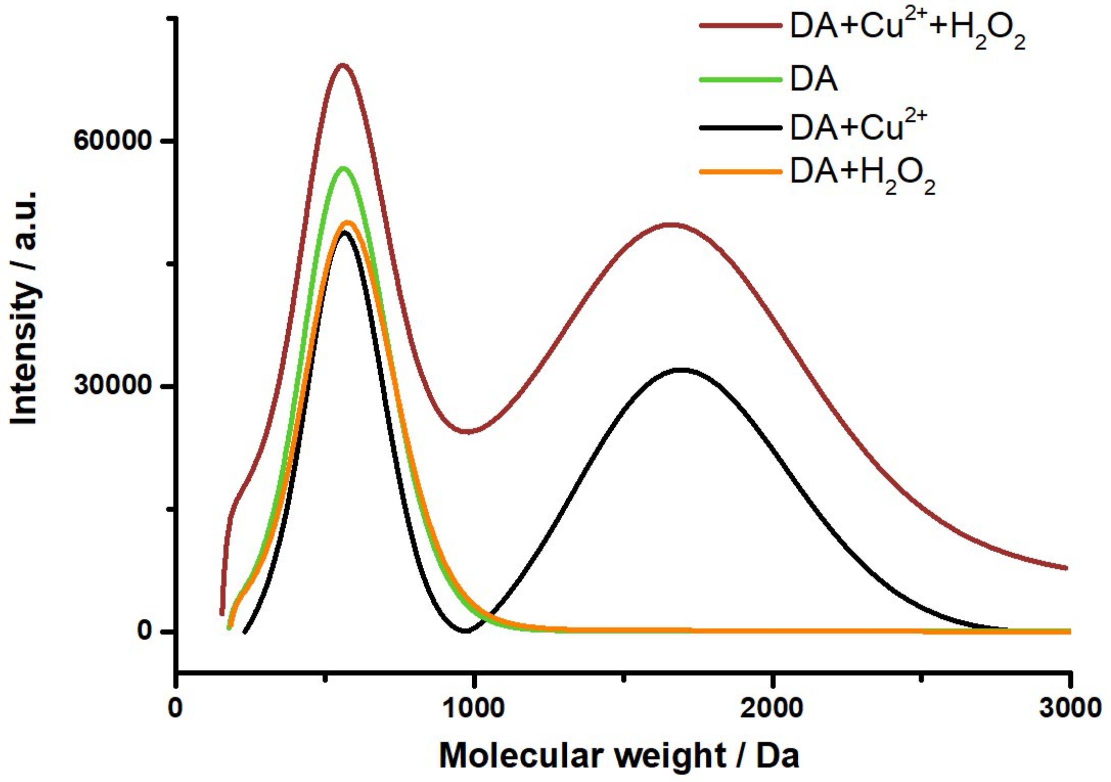

Fig. S1 GPC analysis of DA, DA+Cu^{2+}, DA+H_2O_2, DA+Cu^{2+}+ H_2O_2, respectively.

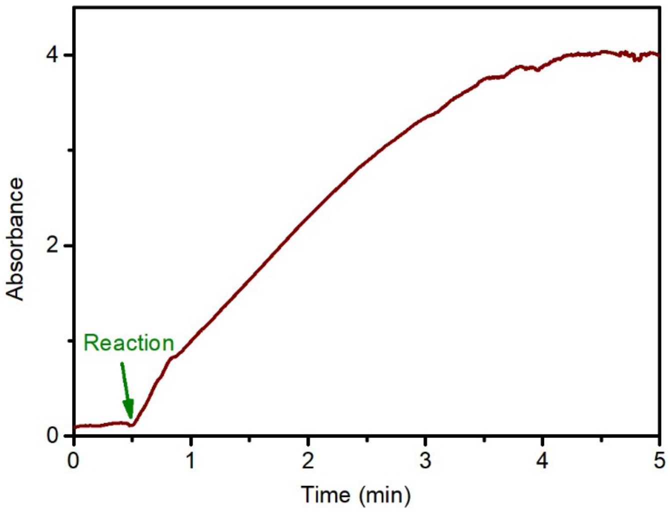

Fig. S2 Reaction kinetics of PDA monitored by UV/Vis spectrophotometer at 465 nm.

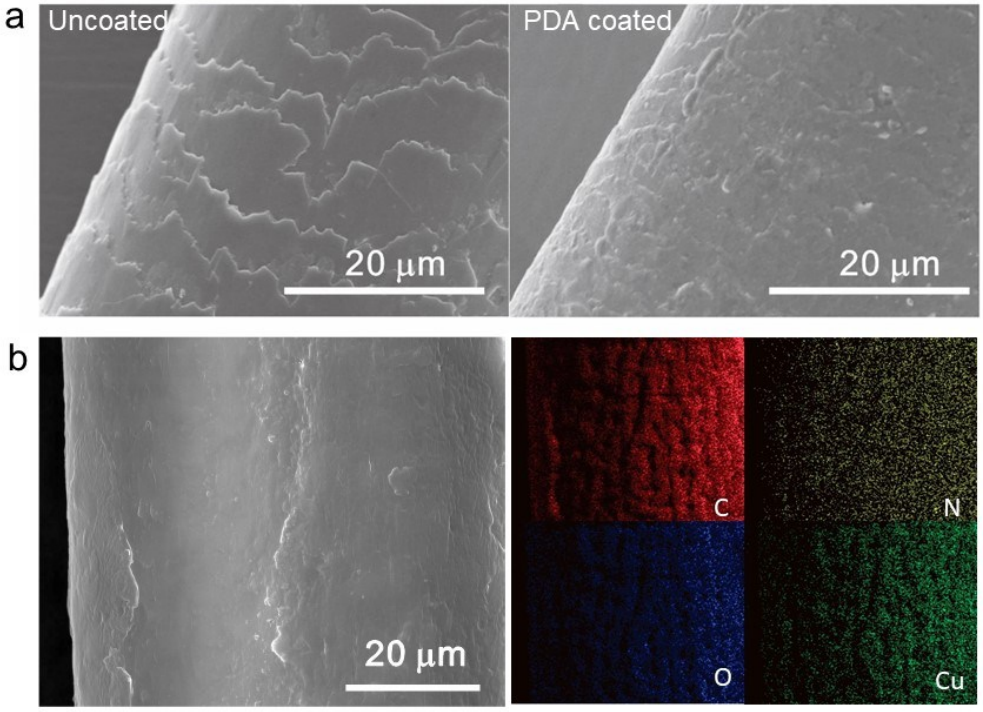

Fig. S3 (a) SEM micrographs of uncoated hair and PDA-coated hair. (b) SEM-EDS mapping of PDA-coated hair.

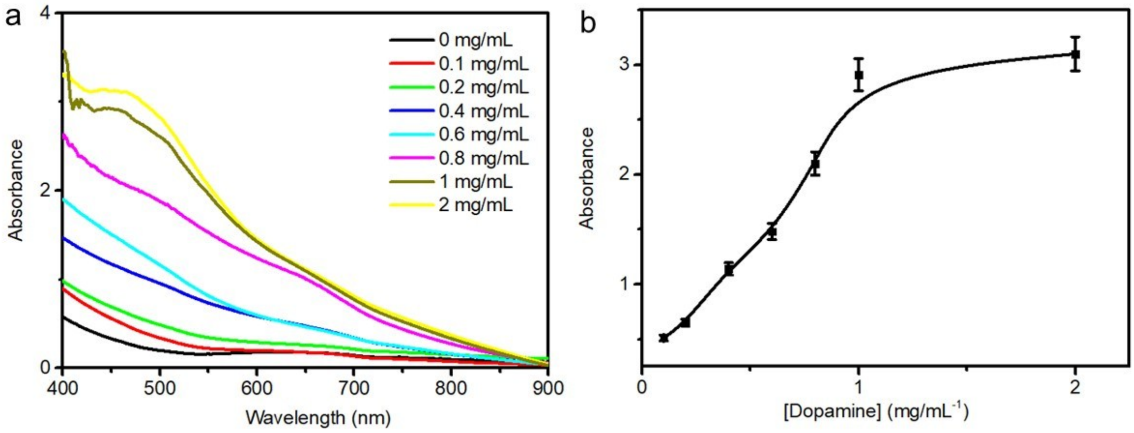

Fig. S4 (a) UV/Vis spectra and (b) absorbances (at 465 nm) of solutions containing 10 mM CuSO4, 15 mM H2O2 and various DA concentrations.

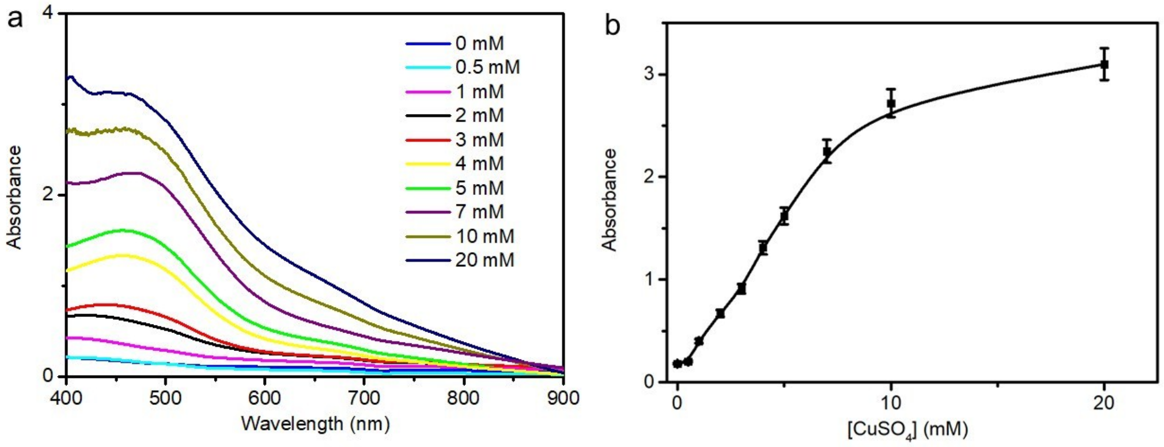

Fig. S5 (a) UV/Vis spectra and (b) absorbances (at 465 nm) of solutions containing 1 mg/mL DA, 15 mM H_2O_2 and various $CuSO_4$ concentrations.

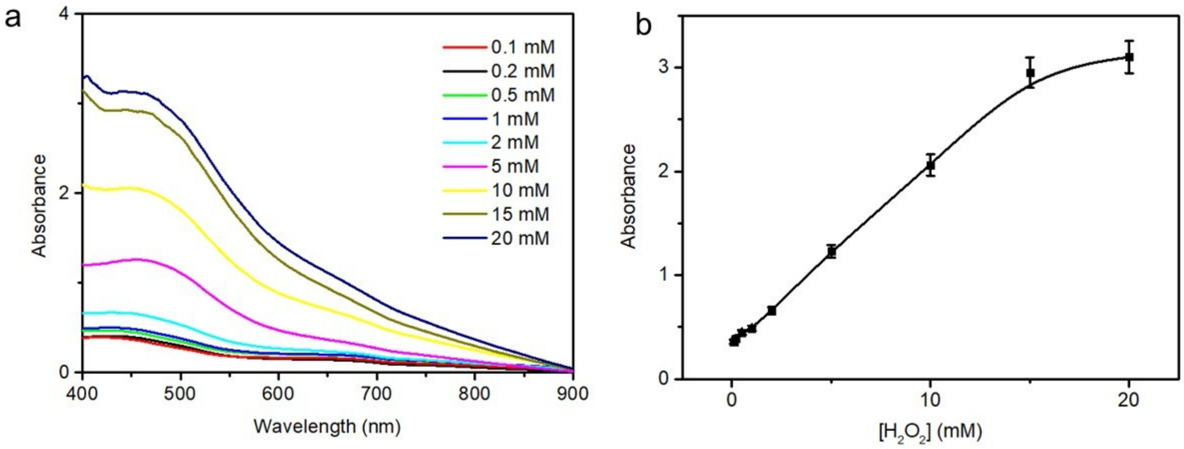

Fig. S6 (a) UV/Vis spectra and (b) absorbances (at 465 nm) of solutions containing 1 mg/mL DA, 10 mM $CuSO_4$ and various H_2O_2 concentrations.

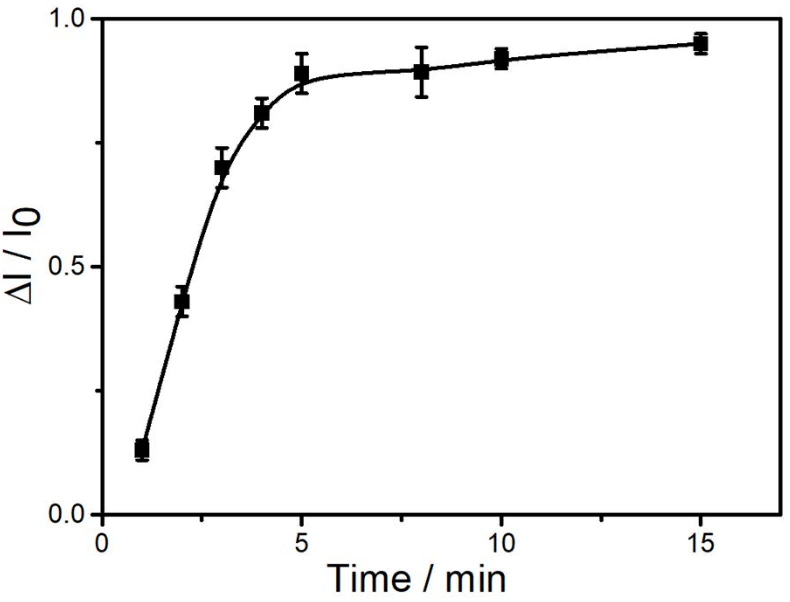

Fig. S7 The diagram of changes of hair color responds to dyeing times.
